# Convenient and Controllable Synthesis of Poly(2-oxazoline)-Conjugated Doxorubicin for Regulating Anti-Tumor Selectivity

**DOI:** 10.3390/jfb14070382

**Published:** 2023-07-21

**Authors:** Min Zhou, Ruxin Cui, Zhengjie Luo, Zihao Cong, Ning Shao, Ling Yuan, Jiawei Gu, Hongyan He, Runhui Liu

**Affiliations:** 1 State Key Laboratory of Bioreactor Engineering, East China University of Science and Technology, Shanghai 200237, China; 2 Key Laboratory for Ultrafine Materials of Ministry of Education, School of Materials Science and Engineering, East China University of Science and Technology, Shanghai 200237, China; 3 Shenzhen Research Institute, East China University of Science and Technology, Shenzhen 518063, China

**Keywords:** poly(2-oxazoline), poly(2-oxazoline)-doxorubicin conjugation, facile synthesis, anti-tumor selectivity

## Abstract

Polyethylene glycol (PEG)–doxorubicin (DOX) conjugation is an important strategy to improve toxicity and enhance clinically therapeutic efficacy. However, with the frequent use of PEG-modified drugs, the accumulation of anti-PEG antibodies has become a tough issue, which limits the application of PEG–drug conjugation. As an alternative solution, poly(2-oxazoline) (POX)−DOX conjugation has shown great potential in the anti-tumor field, but the reported conjugation process of POX with DOX has drawbacks such as complex synthetic steps and purification. Herein, we propose a convenient and controllable strategy for the synthesis of POX−DOX conjugation with different chain lengths and narrow dispersity by *N*-boc-2-bromoacetohydrazide-initiated 2-ethyl-oxazoline polymerization and the subsequent deprotection of the *N*-Boc group and direct reaction with DOX. The DOX−PEtOx conjugates were firstly purified, and the successful conjugations were confirmed through various characterization methods. The synthetic DOX−PEtOx_n_ conjugates reduce the toxicity of DOX and increase the selectivity to tumor cells, reflecting the promising application of this POX−DOX conjugation strategy in drug modification and development.

## 1. Introduction

PEG–drug conjugation is an important technology that is widely used in drug development and clinical application due to numerous functionalities such as reducing drug toxicity and improving pharmacokinetics [1,2,3,4,5,6,7,8]. However, the long-term use of PEG-modified drugs leads to the continuous production and accumulation of anti-PEG antibodies in the body, which increase the risk of adverse immune reactions [9,10,11]. In addition, the efficacy of PEG modification is closely related to the molecular weight, so the PEG molecular weight requires precise control. Meanwhile, the synthesis process of PEG–drug conjugation is complicated and high cost [12]. These disadvantages strictly limit the actual applications of PEG–drug conjugation. Therefore, it is urgent to develop alternative and effective strategies.

Since the successful polymerization of 2-oxazoline was firstly reported by four different research groups in the 1960s, poly(2-oxazoline)s (POXs) have received widespread attention as a promising biomaterial due to their excellent biocompatibility and stability in protease environments [13,14,15,16]. POXs are mainly prepared by the ring-opening polymerization of 2-oxazoline using electrophilic reagents as initiators, and the resulting poly(2-oxazoline)s with terminal active oxazolium ions also can be functionalized by nucleophilic reagents, which provides a convenient synthetic approach in the application of poly(2-oxazoline)s. It is worth noting that peptide-mimicking poly(2-oxazoline)s have been recently proposed for the first time. These functional poly(2-oxazoline)s with potent activities against drug-resistant bacterial and fungus are developed via mimicking host defense peptides [17,18,19,20,21,22,23,24,25], and it was also reported that the polyamine produced by the hydrolysis of poly(2-oxazoline)s exhibited antibacterial activity [26]. On the other hand, poly(2-ethyl-2-oxazoline) (PEtOx) showed strong hydrophilicity and excellent biocompatibility and low immunogenicity under certain temperature and chain length conditions, demonstrating potential as a PEG substitute and peptide mimetic in drug delivery [27,28] and drug conjugation [29,30,31,32,33,34,35]. In the meantime, PEtOx was also found to be thermoresponsive; the LCST behavior depended on the polymer molar mass, and the solubility of PEtOx in water changed with temperature [36]. It also has been used for the modification of small molecule drugs presenting as polymer–drug conjugates [31,33]. Doxorubicin (DOX) is a commonly used first-line anticancer drug for many types of cancer, but its high toxicity and poor solubility restrict the clinical efficacy [31,37]. POX−DOX conjugation can improve its solubility and reduce its toxicity, serving as an effective strategy to enhance the drug safety and therapeutic efficacy [38]. However, the complex synthesis, purification processes and wide molecular weight distribution reported in the current literature have limited practical applications [39].

In this work, we proposed a convenient and controllable strategy for synthesizing POX−DOX conjugation. By the polymerization of 2-ethyl-oxazoline using *N*-boc-2-bromoacetohydrazide as an initiator and the subsequent deprotection of the *N*-Boc group, the hydrazone-terminated PEtOx_n_ was obtained with narrow dispersity and diverse controllable chain length, which can be easily reacted with DOX to produce POX−DOX conjugates DOX−PEtOx_n_. The experimental results showed that a series of DOX−PEtOx_n_ can reduce the toxicity of DOX to mammalian cells and increased the selectivity to tumor cells, among them DOX−PEtOx_20_ exhibiting the best efficiency. These results demonstrate that it is of great efficiency and potential application of our POX−DOX conjugation strategy.

## 2. Materials and Methods

### 2.1. Materials

All chemical reagents and solvents were used without further purification. Anhydrous dichloromethane (DCM), anhydrous *N,N*-dimethylacetamide (DMAc) and dimethyl sulfoxide (DMSO) were purchased from Sigma-Aldrich. Tetrahydrofuran (THF), ethyl acetate (EtOAc) and other solvents were purchased from Shanghai Titan Technology Co., Ltd (Shanghai, China). 2-Ethyl oxazoline, tert-butyl carbazate, bromoacetyl bromide and doxorubicin hydrochloride were purchased from Adamas-beta^®^. Synthesized chemicals were purified using a SepaBean machine equipped with Sepaflash columns produced by Santai Technologies Inc in China (Changzhou, China). The water used in these experiments was obtained from a Millipore water purification system with a resistivity of 18.2 MΩ.cm. 3-(4,5-Dimethylthiazol-2-yl)-2,5-diphenyltetrazolium bromide (MTT) was purchased from MACKLIN regent, Shanghai. Dulbecco’s modified Eagle medium (DMEM) and Roswell Park Memorial Institute-1640 medium (RPMI-1640) were purchased from Hyclone (Logan, UT, USA). Fetal bovine serum (FBS) was purchased from Biological Industries (Kibbutz Beit-Haemek, Israel). Penicillin–streptomycin solution was bought from Guangzhou Sopo Biological Technology Co., LTD (Guangzhou, China). Human umbilical vein endothelial cell line (HUVEC) and the African green monkey kidney fibroblasts (COS-7) cells were obtained from the Cell Bank of the Chinese Academy of Sciences (Shanghai, China). Murine melanoma cell line (B16) was kindly provided by Professor Zhengwei Mao from the Department of Polymer Science and Engineering, Zhejiang University. Lastly, 25 cm^2^ plastic culture flasks and 96-well tissue culture plates were supplied by Shanghai Titan Technology Co., Ltd.

### 2.2. Measurements

Nuclear magnetic resonance (NMR) spectra were collected on a Bruker spectrometer at 400 MHz using CDCl_3_ as the solvent and at 600 MHz using D_2_O as the solvent. The corresponding chemical shifts are referenced to residual protons in the deuterated NMR solvents. High-resolution electrospray ionization time-of-flight mass spectrometry (HRESI-MS) was collected on a Waters XEVO G2 TOF mass spectrometer. Gel permeation chromatography (GPC) was performed on a Waters GPC instrument equipped with a refractive index detector (Waters 2414) using dimethylformamide (DMF), supplemented with 0.01 M LiBr, as the mobile phase at a flow rate of 1 mL/min at 50 °C. The GPC was equipped with a Tosoh TSKgel Alpha-2500 column (particle size 7 µm) and a Tosoh TSKgel Alpha-3000 column (particle size 7 µm) linked in series. The relative number-average molecular weight (*M*_n_), degree of polymerization (DP) and dispersity index (*Ð*) were calculated from a calibration curve using polymethylmethacrylate (PMMA) as standards. Before GPC characterization, all samples were filtered through 0.22 μm polytetrafluoroethylene (PTFE) filters. High-performance liquid chromatography (HPLC) analysis was performed with a SHIMADZU LC 20AR HPLC System equipped with a Luna Omega 5 μm polar C18 column. Purification of the conjugates was performed by preparative HPLC using a SHIMADZU LC 20AR HPLC System equipped with a Luna 5 μm polar C18(2) column. Before purification and analysis, all samples were filtered through 0.22 μm polytetrafluoroethylene (PTFE) filters. Matrix-assisted laser desorption/ionization time-of-flight mass spectrometry (MALDI-TOF-MS) experiments were conducted using an AB SCIEX 4800plus MALDI-TOF analyzer in reflection mode equipped with a nitrogen laser emitting at 355 nm. The 2,5-dihydroxybenzoic acid (DHB) was used as the matrix at the concentration of 20 mg/mL dissolved in acetonitrile (MeCN). All samples were dissolved in MeCN at the concentration of 10 mg/mL. Fourier transform infrared (FT-IR) spectra were collected on a Thermo Electron Nicolet IS50 using KBr as the sample holder. For each spectrum, 32 scans were collected in the 500–4000 cm^−1^ range. The hydrodynamic diameters were measured by dynamic light scattering (DLS) using a Zetasizer Nano-ZS instrument, model ZEN3600 (Malvern Instruments Ltd., Malvern, UK). The samples were filtered before measurement through a 0.8 µm polyethersulfone (PES) filter. The optical density (OD) value and fluorescence value were recorded on a multifunction microplate reader (SpectraMax M2).

### 2.3. Synthesis of N-Boc-2-bromoacetohydrazide

**Scheme 1:**
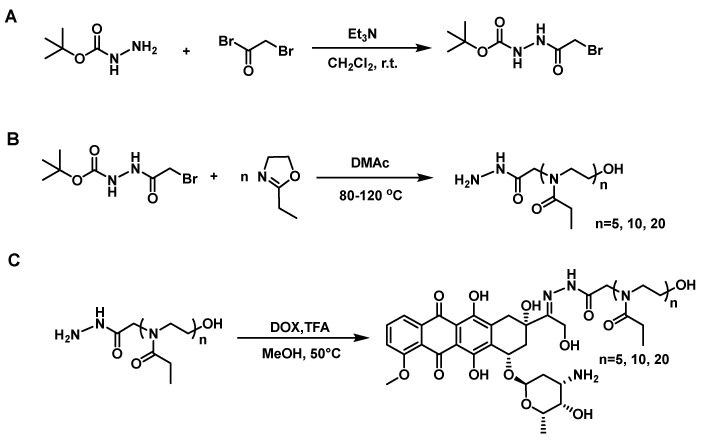
(**A**) Synthesis of *N*-boc-2-bromoacetohydrazide; (**B**) Synthetic route of hydrazide poly(2-ethyl-oxazoline)s; (**C**) Conjugation of doxorubicin and PEtOx.

*N*-boc-2-bromoacetohydrazide was synthesized according to the method in the previous literature [40], as shown in Scheme 1A. To a solution of t-butyl carbazate (5 g, 37.8 mmol, 1.0 equiv.) and triethylamine (5.3 mL, 37.8 mmol, 1.0 equiv.) in dichloromethane (300 mL), bromoacetyl bromide (2.9 mL, 39.7 mmol, 1.1 equiv.) was added dropwise at 0 °C. The reaction mixture was stirred overnight at room temperature under N_2_ atmosphere. The reaction procedure was monitored by thin-layer chromatography (TLC). The mixture was washed with DI water (300 mL × 3), and then the organic layer was dried over anhydrous MgSO_4_. After removing the solvent under vacuum, the crude product was purified by silica gel column chromatography using DCM/MeOH as mobile phases to afford white solid (3.5 g, 45.0% yield). ^1^H NMR (400 MHz, CDCl_3_): δ 3.92 (s, 2H), 1.49 (s, 9H). The obtained ^1^H NMR spectrum was consistent with the report in the precedent literature [40]. HRESI-MS: m/z calculated for [M+ Na]^+^: 275.0; Found 275.0.

### 2.4. Polymerization of 2-Ethyl-oxazoline

**Figure 1:**
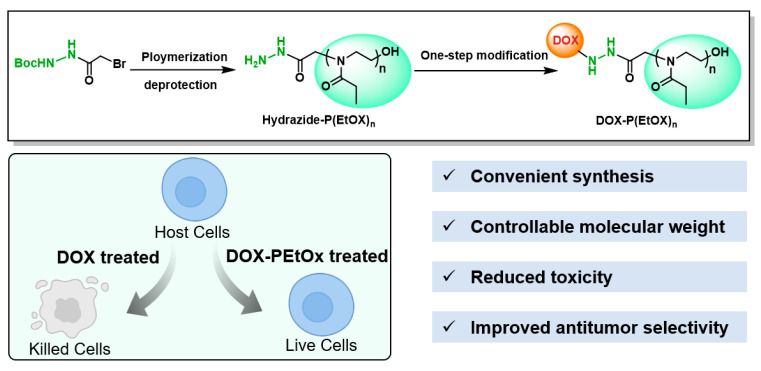
Schematic illustration of the design and drug modification property of DOX−P(EtOx)n. Host cells were killed when treated with free DOX, whereas the cells remained alive after treatment with DOX−PEtOx conjugates.

All homopolymerizations of 2-ethyl-2-oxazoline were carried out under nitrogen atmosphere, and the polymerization reaction is shown in Scheme 1B. The initiator (*N*-boc-2-bromoacetohydrazide) and monomer (EtOx) were dissolved in dry *N,N*-dimethylacetamide (DMAc) to a concentration of 1 M and 2 M, respectively. The degree of polymerization was controlled by the ratio of EtOx: initiator (M:I). For M: I = 5:1, 200 μL of the initiator solution was added to 1.0 mL of the EtOx solution, and the mixture was stirred at 80 °C for 6 h. For M: I = 10:1 and 20:1, 100 μL and 50 μL of the initiator solution were added to 1.0 mL of the EtOx solution, respectively, and the mixtures were stirred at 100 °C for 6 h. When the polymerization reaction was completed, the reaction mixture was cooled to room temperature. The cold methyl tert-butyl ether (MTBE, 45 mL) was poured into the remaining reaction mixture to precipitate out the crude product as a white solid, which was followed by centrifugation to remove the solvent. Then, 2 mL of tetrahydrofuran (THF) was added to resolve the mixture followed by pouring cold methyl tert-butyl ether (MTBE, 45 mL) to precipitate out the crude product. The *N*-Boc-protected polymers were continuously purified by a repeated dissolution–precipitation process using THF/MTBE (2 mL/45 mL) three times to give a white solid. The degree of polymerization (DP) was calculated by ^1^H NMR. The number-average molecular weight (*M*_n_) and dispersity index (*Đ*) were characterized by GPC. The characterizations of polymers are summarized in Figure 1 below.

### 2.5. Synthesis of Deprotected PEtOx

Taking the deprotection of *N*-Boc-protected PEtOx_5_ as an example, a typical procedure is described below. *N*-Boc-protected PEtOx_5_ was dissolved in trifluoroacetic acid (TFA, 2 mL), and then, the reaction solution was kept under shaking for 2 h at room temperature. After the solvent was removed under a nitrogen flow, the residue was dissolved in methanol (1 mL), followed by the addition of cold MTBE (45 mL) to precipitate out the crude polymer. After centrifugation to remove the solvent and drying under vacuum, the crude polymer was collected. After three cycles of the dissolution–precipitation process using methanol/MTBE (1 mL/45 mL), the purified polymer was collected as a yellow solid. After removing the organic solvent, the solid was dissolved in Milli-Q water and subjected to lyophilization to give a yellow powder in the form of TFA salt. The deprotected PEtOx was directly used for further conjugation.

### 2.6. Conjugation of Doxorubicin and PEtOx

Taking the conjugation of PEtOx_5_ and doxorubicin hydrochloride as an example, a typical procedure is described below, as shown in Scheme 1C. PEtOx_5_ (20 mg, 1.0 equiv.) and doxorubicin hydrochloride (26 mg, 1.5 equiv.) were dissolved in 5 mL of dry methanol. After stirring for 10 min, trifluoroacetic acid (10 μL) was added as a catalyst. The reaction mixture was refluxed at 50 °C for 48 h in the dark. The equivalent ratio of polymers and doxorubicin hydrochloride remained the same, while different chain lengths referred to different molecular weight. As for the other two polymers, PEtOx_10_ (30 mg, 1.0 equiv.) with doxorubicin hydrochloride (22 mg, 1.5 equiv.) and PEtOx_20_ (30 mg, 1.0 equiv.) with doxorubicin hydrochloride (12 mg, 1.5 equiv.) were dissolved and reacted following the same procedure.

### 2.7. Purification and Characterization of DOX-PEtOx Conjugates

When the conjugation reactions were completed, the mixtures were cooled to room temperature. After removing the solvent under vacuum, the red solids were resolved with methanol to a concentration of 10 mg/mL. The compounds were purified by preparative RP-HPLC using SHIMADZU LC 20AR HPLC System equipped with a Luna 5 μm polar C18(2) column. All three samples were purified using the same column and method. The purification methods and analytical methods are described below. The mobile phases were water with 0.1% TFA (phase A) and acetonitrile with 0.1% TFA (phase B). Preparative RP-HPLC: 20–80% B for 8 min; 80% B for 7 min; 80–20% B for 8 min. The flow rate of the mobile phase is 10 mL/min. The desired product eluted at 5–7 min and was detected at the wavelengths of 220 nm and 490 nm. The elution was subjected to lyophilization to give a red powder. The chemical bound and *m*/*z* of the products were characterized by FT-IR and MALDI-TOF-MS. The purity of the compound was analyzed by analytical RP-HPLC: 20–80% B for 8 min, 80% B for 7 min, and 80–20% B for 8 min, with a 1 mL/min flow rate, detected at the wavelengths of 220 nm and 490 nm.

### 2.8. Self-Assembling Test of DOX-PEtOx Conjugate

A self-assembling test of the DOX-PEtOx_20_ conjugate was prepared by a similar method [39]. Briefly, 250 μg of DOX-PEtOx_20_ was dissolved in 1 mL of Mili-Q water at 50 °C and then vortexed for 5 min in the dark. The self-assembling ability of the DOX-PEtOx_20_ conjugate was characterized by DLS. The DLS measurements were performed with a scattering angle of 90° at 25 °C. The experiments were carried out with three replicates. Furthermore, the self-assembling ability of the DOX-PEtOx_20_ conjugate was observed by the Tyndall effect.

### 2.9. In Vitro Cytotoxicity Assessment

The in vitro cytotoxicity assessment was conducted according to previous reports with a little change [41]. B16 cells were cultured in RPMI-1640 medium containing FBS (10%) and penicillin/streptomycin (1%). COS-7 and HUVEC cells were incubated in Dulbecco’s modified Eagle’s medium (DMEM) containing FBS (10%) and penicillin/streptomycin (1%). All cells were cultured at 37 °C in a humidified atmosphere containing 5% CO_2_. The cytotoxicity of conjugates was studied using MTT assay. Cells were seeded in 96-well plates at 5000 cells in 100 μL of DMEM or RPMI 1640 medium for each well, and the plates were incubated at 37 °C in a humidified atmosphere containing 5% CO_2_ for 24 h. Different concentrations of DOX-PEtOx solution ranging from 100 to 3.13 μg/mL were prepared and added to HUVEC and COS-7 cells. The plates were incubated for another 48 h. The concentrations of DOX solution ranged from 1 to 0.03 μg/mL. For the cytotoxicity assay of conjugates toward cancer cell B16, the concentrations of DOX-PEtOx_5_ and DOX-PEtOx_10_ ranged from 10 to 0.3 μg/mL, and DOX-PEtOx_20_ solutions ranged from 50 to 1.6 μg/mL, respectively. Untreated cells were used as the negative control.

An aliquot of 10 μL MTT solution (5 mg/mL) in phosphate-buffered saline (PBS) was added in each well, and the plate was incubated for 4 h. After the supernatant was removed, 150 μL DMSO was added in each well, and then the plate was shaken for 15 min before measuring the absorbance at 570 nm. The percentage of cell viability was calculated from cell viability (%) = [(A_polymer_ − A_blank_)/(A _positive control_ − A_blank_) × 100]. The data points of different cell viability and the corresponding polymer concentration were fitted into a curve by using graphpad prism software, and the IC_50_ value was obtained from the fitting curve. All experiments were carried out with three replicates. Each experiment was repeated at least twice on different days.

## 3. Results and Discussion

### 3.1. Synthesis and Characterization of Hydrazide End-Functional PEtOx

**Figure 2:**
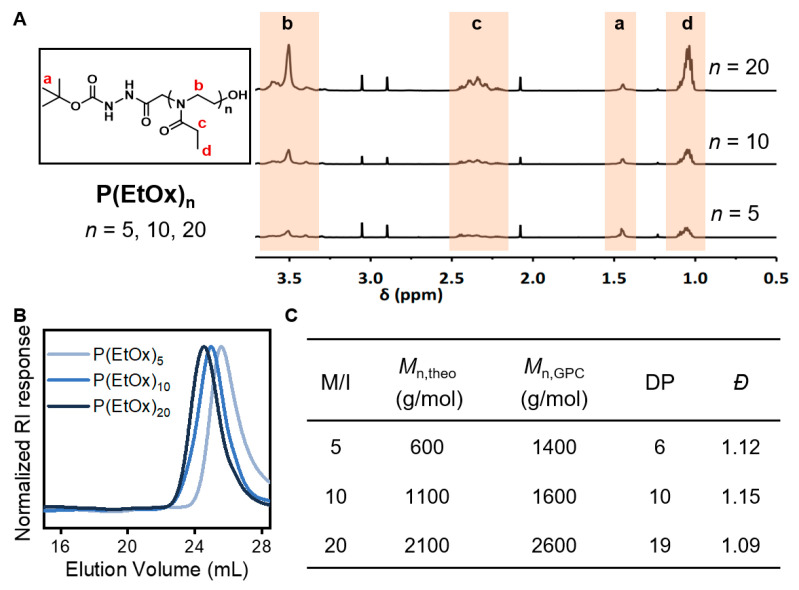
(**A**) ^1^H NMR characterization of protected polymers (D_2_O as solvent, 600 MHz), a, b, c and d represented the characteristics of H at different positions in the chemical structure of the polymers, the ^1^H NMR spectra was cut to 0.5–3.7 ppm because this range includes the characteristic peaks, and the complete spectra can be found in the Supporting Information; (**B**) GPC traces of PEtOx of three chain lengths; (**C**) GPC characterization of PEtOx of three chain lengths. *M*_n_ and *Đ* were determined by GPC using DMF as the mobile phase at a flow rate of 1 mL/min. DP was determined by ^1^H NMR. *M*_n,theo_ is the theoretical number average molecular weight; *M*_n,GPC_ is the obtained number average molecular weight analyzed by GPC; DP is the obtained degree of polymerization; *Đ* means the dispersity.

A series of PEtOx were synthesized via the cationic ring-open polymerization of 2-ethyl-2-oxazoline using *N*-boc-2-bromoacetohydrazide as an initiator. We conducted ^1^H NMR analysis to determine the chain length of PEtOx. As shown in Figure 2A and Figure S3–S5, a sharp peak at 1.5 ppm corresponding to the methyl protons of Boc and a peak at 1.01 ppm attributed to methyl protons in the side chains were selected. The chain length (DP = 6, 10, 19) determined by ^1^H NMR closely matched our expectations (calculated DP = 5, 10, 20). Moreover, the control of polymer molecular weight is very important and crucial for functionality [42,43,44,45,46]. Notably, when thin-layer chromatography showed full monomer conversion, the resulting polymers exhibited a GPC trace with a single peak and narrow dispersity (*Đ* = 1.09–1.15) (Figure 2B and Figure S6–S8), which indicated a uniform polymer distribution. The obtained DP values analyzed by GPC characterization were consistent with those in ^1^H NMR characterization, reflecting a near match to the ratio of the monomer/initiator (Figure 2C). To encapsulate, the hydrazide end-functional PEtOx was successfully obtained followed by deprotection of the NH-Boc group.

### 3.2. Synthesis and Characterization of DOX-PEtOx Conjugates

**Figure 3:**
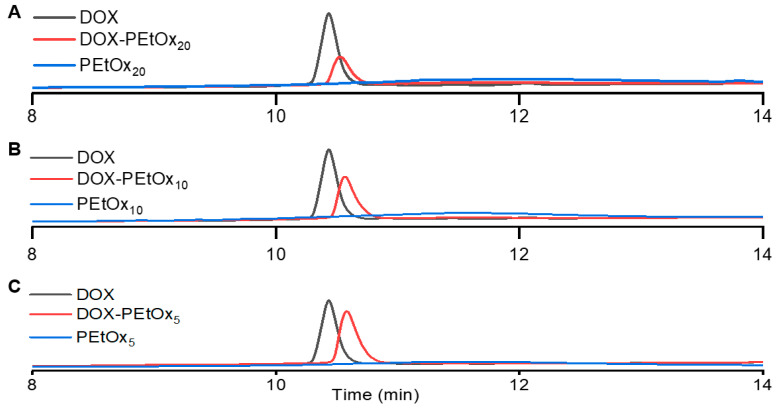
HPLC spectrum of DOX, PEtOx and DOX−PEtOx conjugates: (**A**) Comparation among DOX, PEtOx_5_ and DOX−PEtOx_5_; (**B**) Comparation among DOX, PEtOx_10_ and DOX-PEtOx_10_; (**C**) Comparation among DOX, PEtOx_20_ and DOX-PEtOx_20_. The analysis was performed with a SHIMADZU LC 20AR HPLC System equipped with a Luna Omega 5 μm polar C18 column. All samples (DOX, PEtOx and DOX−PEtOx) were analyzed with the same method: 20–80% phase B for 8 min; 80% phase B for 7 min; 80–20% phase B for 8 min, with 1 mL/min flow rate, detected at the wavelength of 220 nm.

The conjugations of DOX and hydrazide end-functional PEtOx with different chain lengths were conducted as described above. The purity of compounds is one of the critical factors in the field of drug development. However, it largely depended on the synthesis and purification method. In order to characterize the purity of DOX−PEtOx conjugates, we conducted RP−HPLC analysis monitored at a wavelength of 220 nm. The result portrayed in Figure 3 showed a slight change in the elution time of the DOX−PEtOx conjugates compared with free doxorubicin, indicating the difference of the adsorption capacity and the column. On the RP-HPLC spectrum of DOX−PEtOx, a single conspicuous strong peak showed up within 15 min, while no other peaks were present. This monitoring result suggested the high purity of DOX−PEtOx conjugations with all three lengths. The characterizations above unambiguously proved the great convenience and high purity of the preparation method of DOX−PEtOx conjugates.

**Figure 4:**
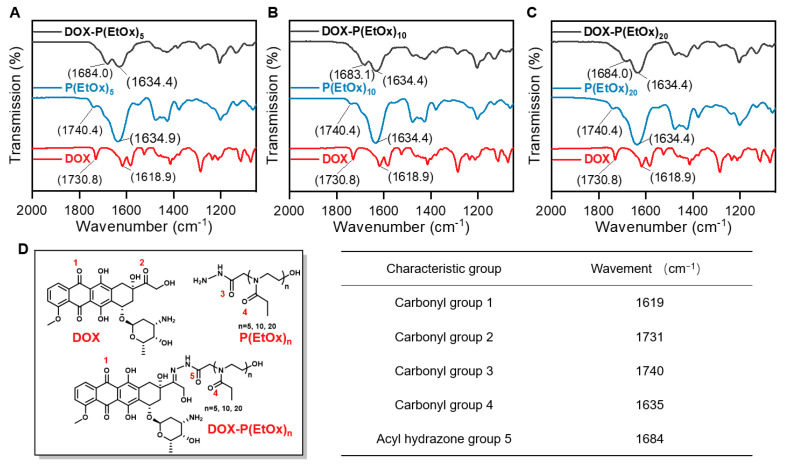
FTIR spectra of DOX, PEtOx and DOX−PEtOx conjugates: (**A**) Comparison among DOX, PEtOx_5_ and DOX−PEtOx_5_; (**B**) Comparison among DOX, PEtOx_10_ and DOX−PEtOx_10_; (**C**) Comparison among DOX, PEtOx_20_ and DOX−PEtOx_20_; (**D**) The summary of the FTIR signals of characteristic groups of DOX and polymers.

The DOX−PEtOx conjugates were further characterized by FTIR spectra using KBr as the sample holder. In spectra of DOX, the peak at 1619 cm^−1^ resulted from the absorption of unreactive carbonyl of the anthracene ring in DOX, and the peak at 1731 cm^−1^ was attributed to the stretching vibration of the reactive carbonyl group of the ketone in DOX. In the spectra of PEtOx, the strong peak at 1635 cm^−1^ is from the carbonyl vibration of the amide groups on the polymer side chain, and the peak at 1740 cm^−1^ belongs to the carbonyl absorption of hydrazide. After conjugation, the characteristic peak (1731 cm^−1^) from the reactive carbonyl group in DOX and the characteristic peak (1740 cm^−1^) from the terminal carbonyl group in the polymer disappeared in all DOX−PEtOx conjugates, whereas the carbonyl absorption peak from the polymer side chain at 1635 cm^−1^ remained. In addition, the peaks at 1684 cm^−1^ and 1635 cm^−1^ in DOX−PEtOx conjugates might be attributed to the acyl hydrazone bond and the amide groups on the polymer side chain, respectively. Moreover, the FTIR spectra of three PEtOx_n_ and corresponding DOX−PEtOx_n_ conjugates with different polymer chain lengths showed the consistent characteristic peaks and identical changes, which also indicated the repeatability of polymer synthesis and DOX modification in our DOX−PEtOx conjugates strategy. These results reflected the successful conjugation of DOX with PEtOx (Figure 4).

**Figure 5:**
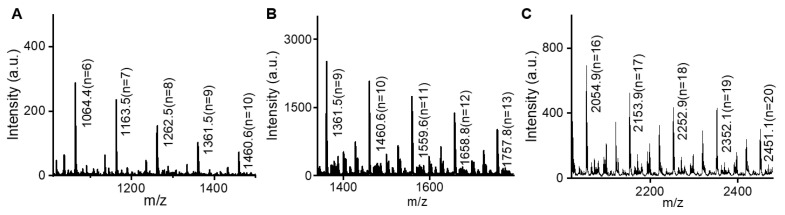
MALDI-TOF MS spectrum of DOX-PEtOx conjugates: (**A**) DOX−PEtOx_5_; (**B**) DOX−PEtOx_10_; (**C**) DOX−PEtOx_20_. The 2,5-dihydroxybenzoic acid (DHB) was used as the matrix dissolved in acetonitrile (MeCN) at a concentration of 20 mg/mL. All conjugates were dissolved in MeCN at the concentration of 10 mg/mL. The matrix and conjugates were mixed with *v/v* 5:1 and used as samples for MALDI-TOF MS characterization. The data were analyzed by data explorer and origin.

The DOX−PEtOx conjugates purified by preparative RP−HPLC were further confirmed by MALDI−TOF−MS characterization. The 99 Da difference between peaks reflected the 2-ethyl-2-oxazoline residues. DOX was reported to be easily ionized (fragment ions at *m*/*z* 397.2) in a positive interface [47,48]. Taking this into consideration, the specific mass corresponding to each peak revealed the successful conjugation of DOX and PEtOx (Figure 5).

### 3.3. In Vitro Cytotoxicity and Anti-Tumor Efficacy of DOX-PEtOx Conjugates

**Figure 6:**
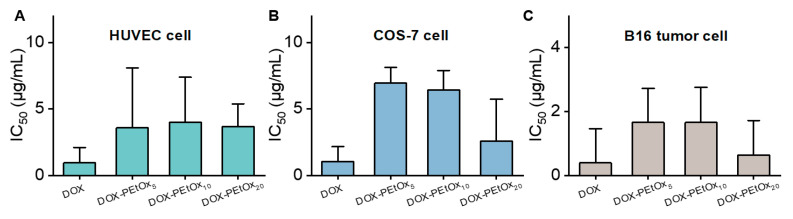
Mammalian cytotoxicity (IC_50_, μg/mL) of three DOX−PEtOx conjugates toward (**A**) HUVEC cells and (**B**) COS-7 cells determined by MTT assay; (**C**) anticancer activity (IC_50_, μg/mL) of conjugates against B16 cells determined by MTT assay. Free DOX was used as control. Three replicates were conducted in each experiment; all the experiments were repeated for twice.

The further in vitro cytotoxicity and anticancer activity of DOX−PEtOx conjugates were evaluated using MTT assay for determining the half-maximal inhibitory concentration (IC_50_). It is noteworthy that DOX−PEtOx conjugates showed lower cytotoxicity against mammalian cells than free DOX (IC_50_ = 0.9 μg/mL toward HUVEC, IC_50_ = 1.0 μg/mL toward COS-7). The IC_50_ values of conjugates range from 3.5 to 4.0 μg/mL against HUVEC and from 2.6 to 7.0 μg/mL against COS-7 (Figure 6A,B). Via the modification with PEtOx, the cytotoxicity of DOX toward mammalian cells was largely reduced by a factor of three to eight. Meanwhile, the DOX−PEtOx conjugates manifested cell growth inhibitory activity against B16 cancer cells (IC_50_ in range of 0.6–1.7 μg/mL) (Figure 6C).

### 3.4. Anti-Tumor Selectivity of DOX-PEtOx Conjugates

**Figure 7:**
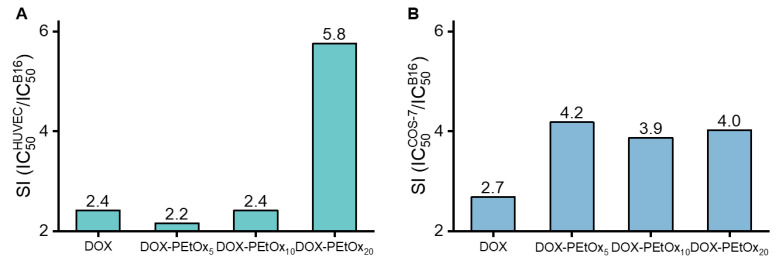
Selectivity index of DOX−PEtOx calculated from: (**A**) IC_50_(HUVEC)/IC_50_(B16); (**B**) IC_50_(COS-7)/IC_50_(B16).

The selectivity index (SI) was calculated and used as a standard to characterize the anti-tumor selectivity of drugs. A higher SI means a higher selectivity toward tumor cells. Noticeably, the SI values (IC_50_ (COS-7)/IC_50_ (B16)) of conjugates with all three lengths were 1.5 times higher than those of free DOX (Figure 7B). Meanwhile, the SI (IC_50_ (HUVEC)/IC_50_ (B16)) of DOX−PEtOx_20_ showed values 2.7 times higher than those of free DOX (Figure 7A). The conjugate with the polymer chain length of 20 displayed the best selectivity when HUEVEC cells are considered, while all DOX−PEtOx conjugates with different polymer chain lengths showed superior selectivity to free DOX. Based on the cytotoxicity and in vitro anticancer activity studies, the conjugates were less toxic and superior in selectivity against cancer cells versus mammalian cells, which may be explained by the modification of PEtOx. The hydrophilicity and charge properties of DOX changed, thereby affecting the interaction between DOX and mammalian cell membranes. It is concluded that via the modification with PEtOx, the anticancer selectivity of DOX was improved, suggesting the great potential use of DOX−PEtOx conjugates as a therapeutic method.

### 3.5. Self-Assembling Ability of DOX-PEtOx Conjugates

**Figure 8:**
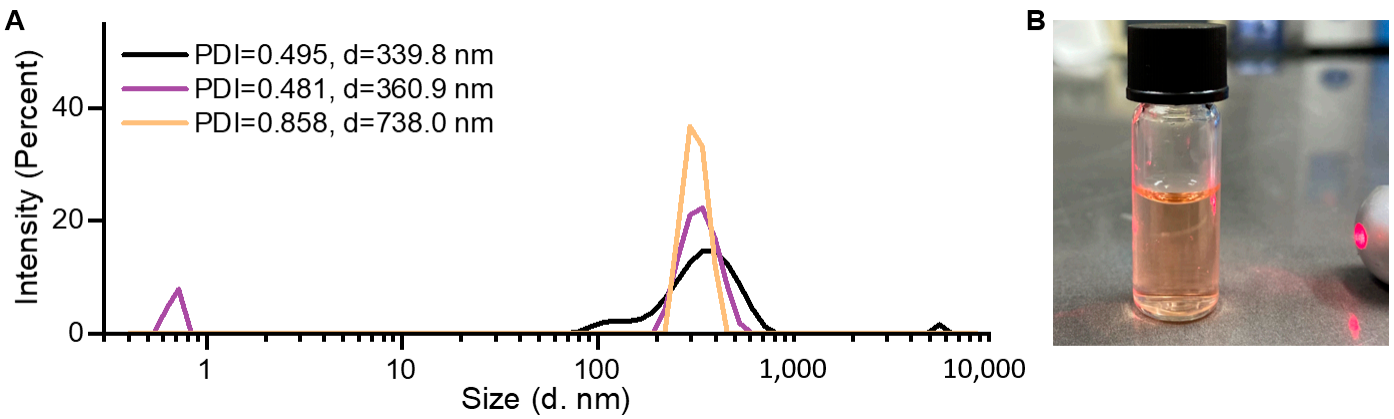
Proof used to form unstable self-assembling micelles at aqueous solution: (**A**) Size distribution of DOX−PEtOx conjugates characterized by DLS measurement thrice; (**B**) Tyndall observation of DOX−PEtOx conjugates.

As previously reported, the DOX-PEOx_20_ with a 1.48 polydispersity index and 2610 g/mol number-average molecular weight was prepared via ethyl 3-bromopropionate initiated 2-ethyl-2-oxazoline polymerization under the presence of potassium iodide and the subsequent four-step post-modification, which resulted in a 10-fold decrease in the in vitro anti-tumor activity compared with free DOX [39]. Differently, DOX−PEOx_20_ with a 1.09 polydispersity index and 2600 g/mol number-average molecular weight was prepared from our synthetic strategy and showed a comparative anti-tumor activity. The different synthesis route and GPC characterization results caused the difference in the anti-tumor activity of DOX−PEOx_20_ conjugates. We further studied the self-assembling behavior of DOX−PEtOx conjugation from our synthetic strategy and multi-step post-polymerization functionalization strategy in the same condition. The three straight DLS measurements of DOX−PEOx_20_ we synthesized showed that the particle size results were completely inconsistent with d values at 339.8, 360.9 and 738.0, and the distribution was very wide and different with PDI values at 0.495, 0.481 and 0.859, indicating that DOX-PEtO_20_ did not form stable assemblies (Figure 8A), which was consistent with the experimental result that no Tyndall effect was observed (Figure 8B). The experiments above demonstrated that the synthetic strategy had a significant impact on the DLS results of DOX−PEOx_20_ conjugates. Despite observing some self-assembling behavior in the DOX−PEOx_20_ conjugates prepared using our strategy, the DLS results varied when compared to DOX−PEOx_20_ conjugates obtained using previously reported synthetic strategies.

## 4. Conclusions

In this work, we demonstrate a new synthetic strategy for the convenient and controllable synthesis of POX−DOX conjugates. By initiating 2-ethyl-oxazoline polymerization with *N*-boc-2-bromoacetohydrazide and subsequent deprotection, we prepared the hydrazone-terminated PEtOx_n_ with varying chain lengths and narrow dispersity, which could be easily conjugated with doxorubicin in a one-step reaction to obtain DOX−PEtOx_n_ conjugates. The convenient method we proposed for synthesizing end-functional poly(2-oxazoline)s has largely shortened the paths and lowered the cost for poly(2-oxazoline)s–drug conjugation. Our synthetic strategy addresses the challenges of complex synthetic steps, difficult purification and broad molecular weight distribution in the currently reported POX−DOX conjugation strategy and realizes the controllable preparation of DOX−PEtOx_n_ conjugates, which could contribute to precisely controlling the molecular weight of polymers for DOX modification and maintaining a stable efficacy. Moreover, we have investigated the effectiveness of the DOX−PEtOx_n_ conjugates against tumor cells B16 along with the cytotoxicity toward mammalian cells HUVEC and COS-7 using MTT assay. Compared to DOX, the DOX−PEtOx_n_ conjugates we synthesized significantly improved the anti-tumor efficacy and selectivity against mammalian cells, of which DOX-PEtOx_20_ displayed the best performance. In summary, our proposed synthesis strategy and synthesized POX−DOX conjugates have promising application potential in drug modification and the biomedical field.

## Data Availability

The data that support this study are available from the corresponding author upon reasonable request.

## Supplementary Materials

The following supporting information can be downloaded at: https://www.mdpi.com/article/10.3390/jfb14070382/s1. Figure S1. ^1^H NMR spectrum of *N*-boc-2-bromoacetohydrazide; Figure S2. HRESI-MS spectrum of *N*-boc-2-bromoacetohydrazide; Figure S3. ^1^H NMR spectrum of PEtOx_5_ in D_2_O; Figure S4. ^1^H NMR spectrum of PEtOx_10_ in D_2_O; Figure S5. ^1^H NMR spectrum of PEtOx_20_ in D_2_O; Figure S6. GPC trace of PEtOx_5_; Figure S7. GPC trace of PEtOx_10_; Figure S8. GPC trace of PEtOx_20_.
