# Supplementary material for: Convenient and Controllable Synthesis of Poly(2-oxazoline)-Conjugated Doxorubicin for Regulating Anti-Tumor Selectivity
Source: J Funct Biomater. 2023 Jul 21;14(7):382. doi: 10.3390/jfb14070382 (PMC10381835; doi:10.3390/jfb14070382)
Supplement: Supplementary file 1 [file jfb-14-00382-s001.zip › jfb-2460634-supplementary.pdf]

Convenient and Controllable Synthesis of Poly(2-oxazoline)-Conjugated Doxorubicin for Regulating anti-Tumor Selectivity

Min Zhou,[*,†,1] Ruxin Cui, [†, 2] Zhengjie Luo,[2] Zihao Cong[2], Ning Shao,[2] Ling Yuan,[2] Jiawei Gu,[2] Hongyan He,[2] and Runhui Liu[*,1,2,3]

[1]State Key Laboratory of Bioreactor Engineering, East China University of Science and Technology, Shanghai 200237, China

[2]Key Laboratory for Ultrafine Materials of Ministry of Education, School of Materials Science and Engineering, East China University of Science and Technology, Shanghai 200237, China

[3]East China University of Science and Technology Shenzhen Research Institute, Shenzhen 518063, China

[†]These authors contribute equally to this work.

Correspondence should be addressed to M.Z. (minzhou@ecust.edu.cn), R.L. (rliu@ecust.edu.cn).

Supplementary Materials

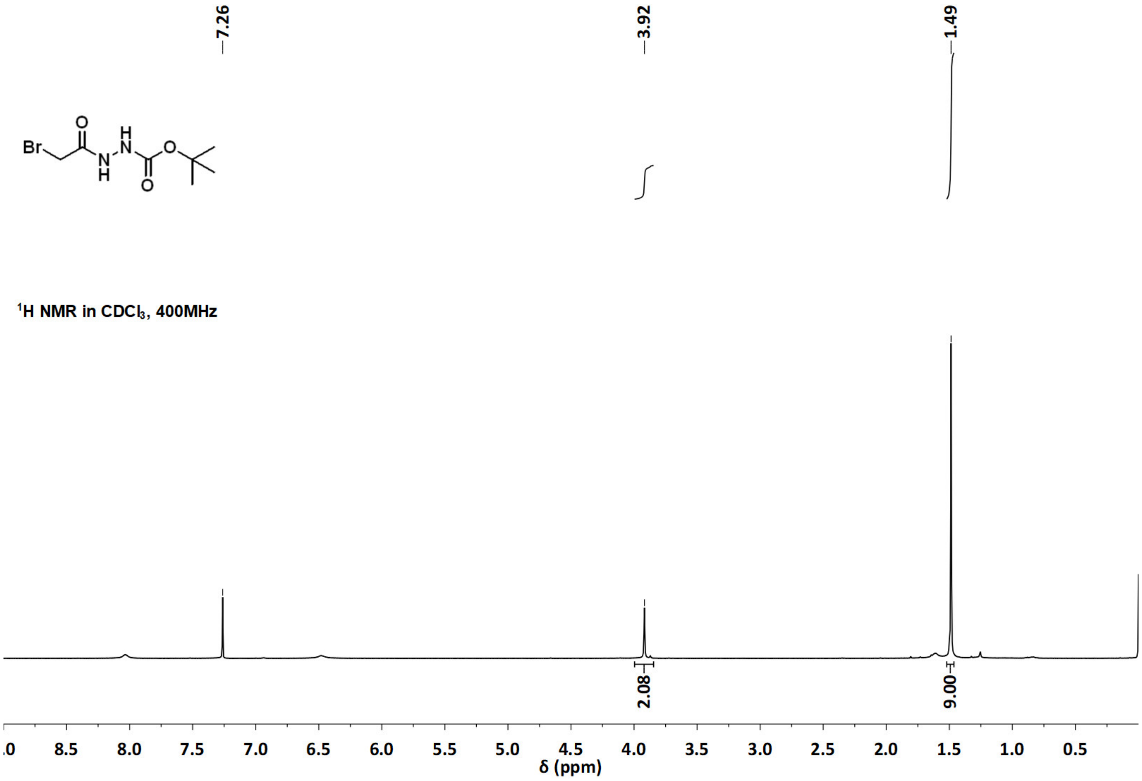

Figure S1. ^{1}H NMR spectrum of *N*-boc-2-bromoacetohydrazide.

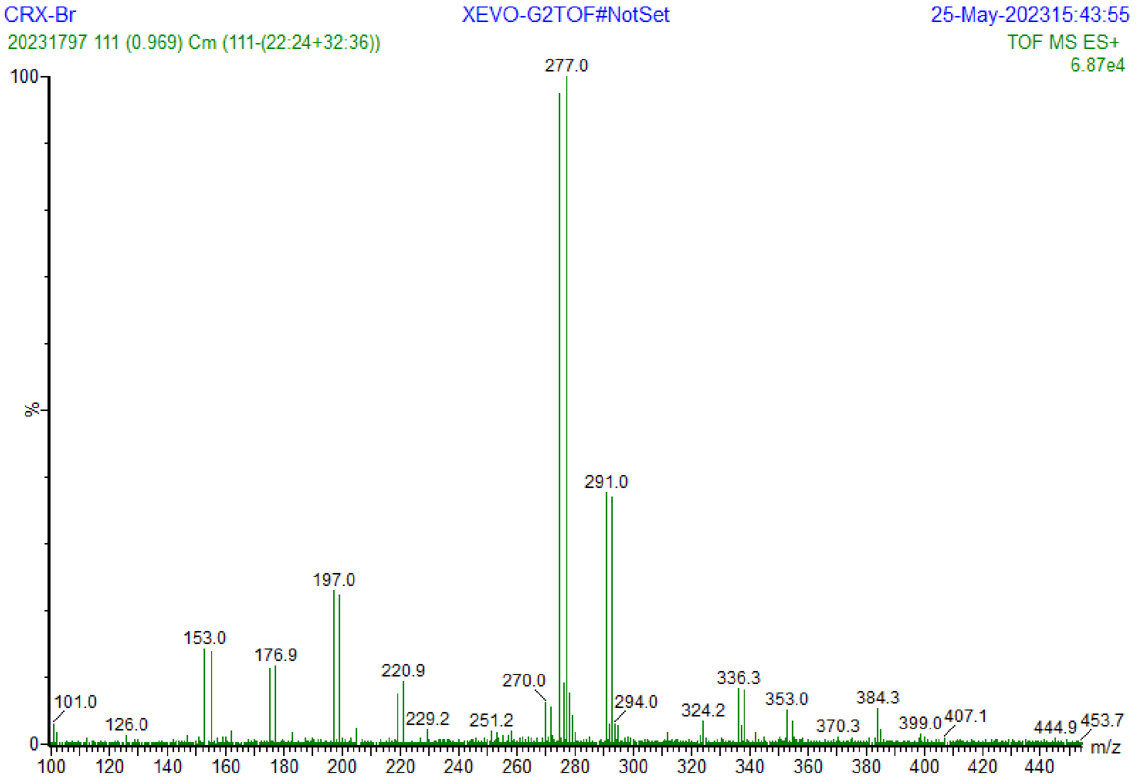

Figure S2. ESI-MS spectrum of N-boc-2-bromoacetohydrazide.

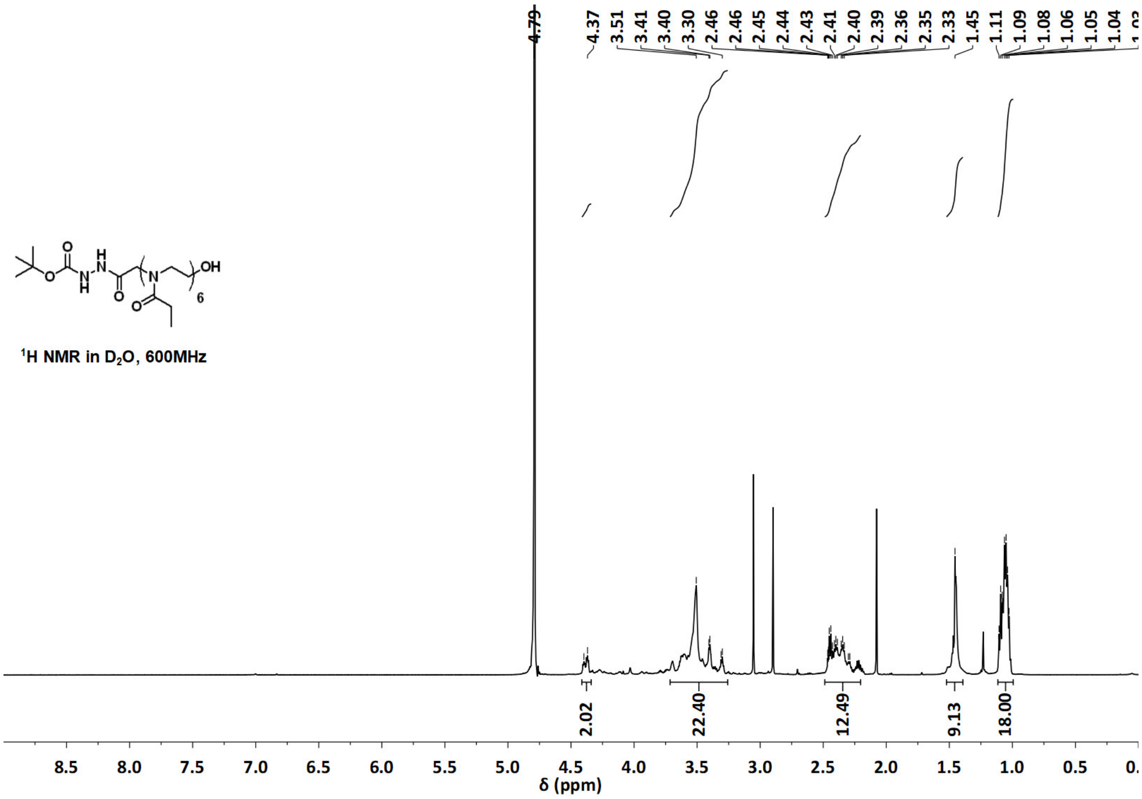

Figure S3. ^{1}H NMR spectrum of PEtOx$_5$ in D_2O.

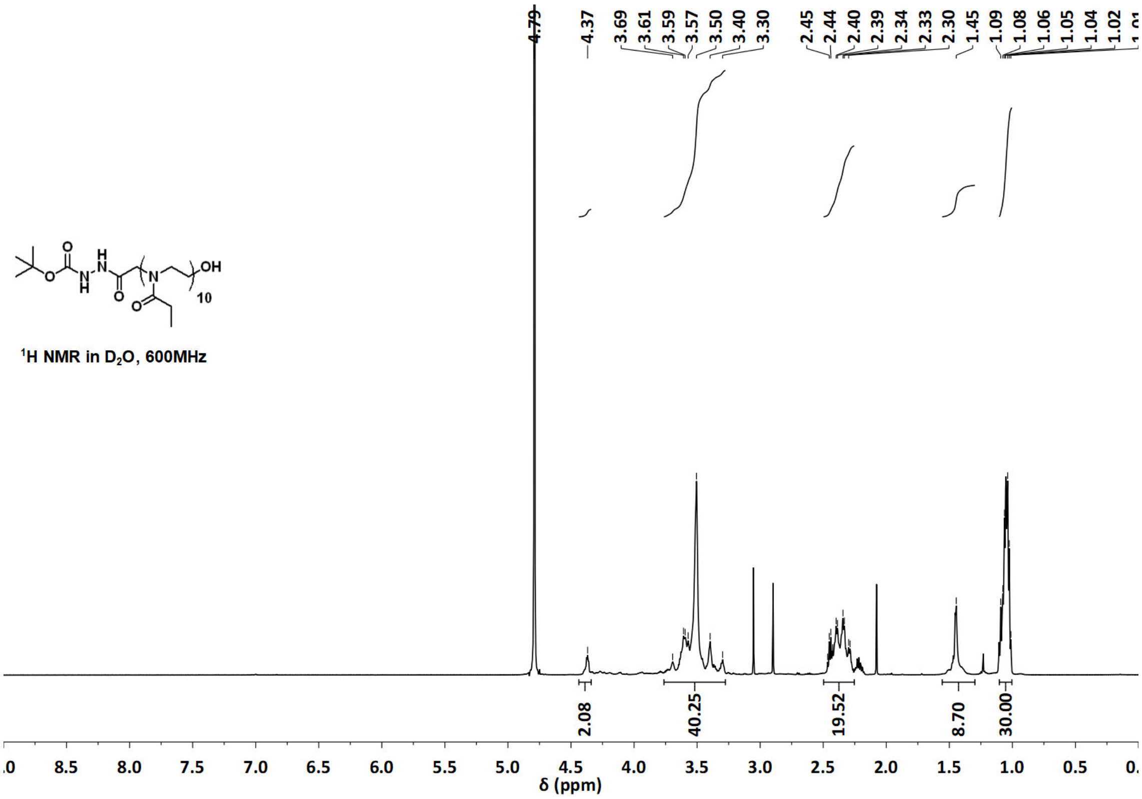

Figure S4. ^{1}H NMR spectrum of PEtOx$_{10}$ in D_2O.

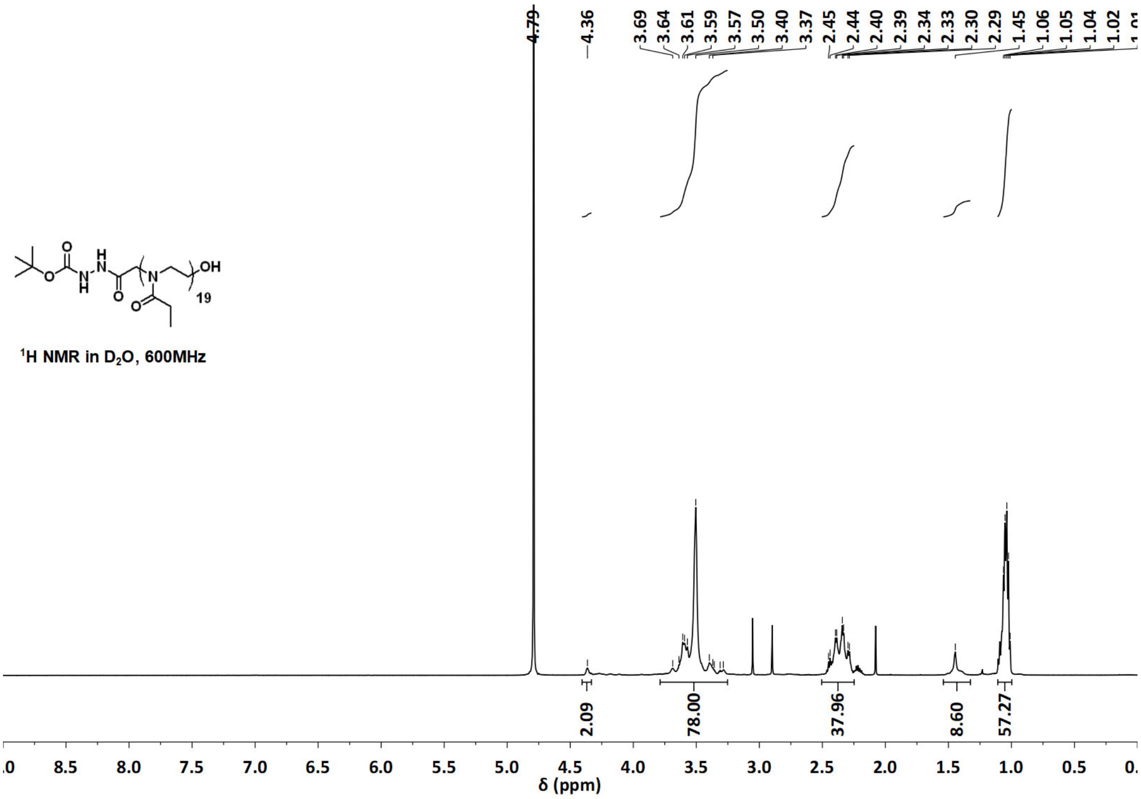

Figure S5. ^{1}H NMR spectrum of $PEtOx_{20}$ in D_2O.

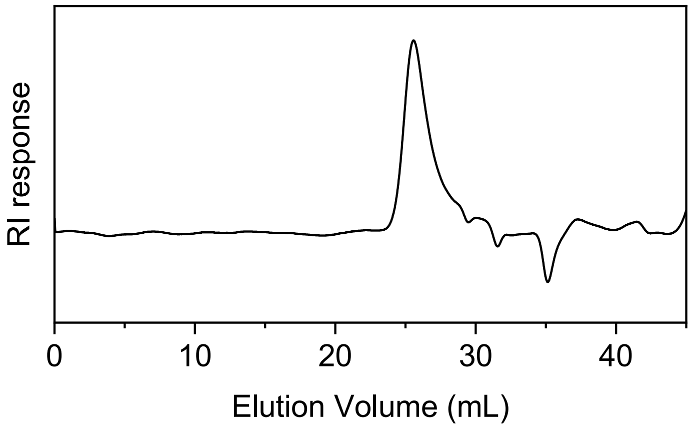

Figure S6. GPC trace of $PEtOx_5$, DMF was used as the mobile phase at a flow rate of 1 mL/min.

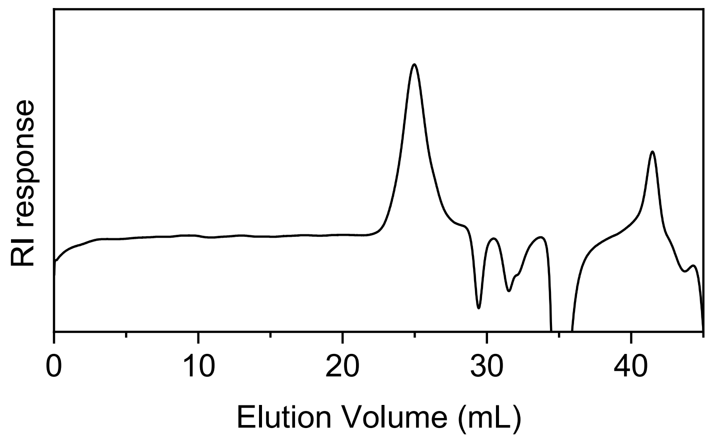

Figure S7. GPC trace of $PEtOx_{10}$, DMF was used as the mobile phase at a flow rate of 1 mL/min.

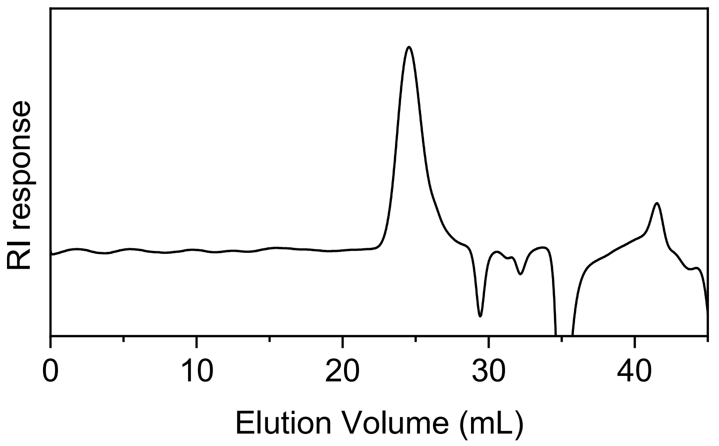

Figure S8. GPC trace of $PEtOx_{20}$, DMF was used as the mobile phase at a flow rate of 1 mL/min.
